# Alleviation of Cadmium Toxicity in *Brassica juncea* L. (Czern. & Coss.) by Calcium Application Involves Various Physiological and Biochemical Strategies

**DOI:** 10.1371/journal.pone.0114571

**Published:** 2015-01-28

**Authors:** Parvaiz Ahmad, Maryam Sarwat, Nazir Ahmad Bhat, Mohd Rafiq Wani, Alvina Gul Kazi, Lam-Son Phan Tran

**Affiliations:** 1 Department of Botany, S. P. College, Srinagar-190001, Jammu and Kashmir, India; 2 Amity Institute of Pharmacy, Amity University, NOIDA, 201303, India; 3 College of Applied Medical Sciences, Prince Salman bin Abdul Aziz University, Al-Kharj, Riyadh, 11942, Kingdom of Saudi Arabia; 4 Department of Botany, Government Degree College (Boys), Anantnag-192102, Jammu and Kashmir, India; 5 Atta-ur-Rehman School of Applied Biosciences, National University of Science and Technology, H-12, Islamabad, 44000, Pakistan; 6 Signaling Pathway Research Unit, RIKEN Center for Sustainable Resource Science, 1-7-22, Suehiro-cho, Tsurumi, Yokohama, 230-0045, Japan; Institute of Genetics and Developmental Biology, Chinese Academy of Sciences, CHINA

## Abstract

Calcium (Ca) plays important role in plant development and response to various environmental stresses. However, its involvement in mitigation of heavy metal stress in plants remains elusive. In this study, we examined the effect of Ca (50 mM) in controlling cadmium (Cd) uptake in mustard (*Brassica juncea* L.) plants exposed to toxic levels of Cd (200 mg L^−1^ and 300 mg L^−1^). The Cd treatment showed substantial decrease in plant height, root length, dry weight, pigments and protein content. Application of Ca improved the growth and biomass yield of the Cd-stressed mustard seedlings. More importantly, the oil content of mustard seeds of Cd-stressed plants was also enhanced with Ca treatment. Proline was significantly increased in mustard plants under Cd stress, and exogenously sprayed Ca was found to have a positive impact on proline content in Cd-stressed plants. Different concentrations of Cd increased lipid peroxidation but the application of Ca minimized it to appreciable level in Cd-treated plants. Excessive Cd treatment enhanced the activities of antioxidant enzymes superoxide dismutase, ascorbate peroxidase and glutathione reductase, which were further enhanced by the addition of Ca. Additionally, Cd stress caused reduced uptake of essential elements and increased Cd accumulation in roots and shoots. However, application of Ca enhanced the concentration of essential elements and decreased Cd accumulation in Cd-stressed plants. Our results indicated that application of Ca enables mustard plant to withstand the deleterious effect of Cd, resulting in improved growth and seed quality of mustard plants.

## Introduction

Heavy metal stress has been increasing at an alarming rate and has become a global issue in the contemporary era. Heavy metal pollution in soil arises as a result of various anthropogenic activities, such as continuous use of sewage water, sewage sludge and fertilizers [1–2]. Approximately, 60% of the cultivated soils have globally mineral problems [3], such as metal toxicities along with nutritional and metal deficiencies [4–5]. Heavy metals have been reported to inhibit the growth and productivity of crops [6–7]. Among them, cadmium (Cd) is well known noxious environmental element due to its great toxicity and high mobility from soil to plants [8].

Cd is naturally occurring element and has been detected in more than 1,000 species of aquatic and terrestrial flora and fauna. The release of Cd from antropogenic activities has been estimated to be about 4,000–13,000 tons per year [9–10]. Being a highly toxic metal pollutant of soil, Cd inhibits root and shoot growth, compresses yield production and is frequently accumulated by agriculturally important crops, thus entering the food chain with a significant potential to impair human and animal health [11]. Many people suffer from renal tubular disease by consuming rice cultivated on high Cd-polluted soils [12]. Excessive amount of Cd may cause decreased uptake of nutrient elements and chlorophyll (Chl) degradation as well as various morphological and biochemical parameters [11,13]. Disturbance in the plant water relations is widely known as one of the first harmful effects of Cd toxicity [14], which leads to osmotic stress in plants.

Plants accumulate low molecular mass compounds, the compatible solutes that do not interfere with the normal biochemical reactions rather they replace water in biochemical reactions [15]. Overproduction of different types of compatible solutes during Cd stress is an important biochemical response of plants [13,16]. Cd is responsible for induction of oxidative stress through generation of reactive oxygen species (ROS). These ROSs are deleterious as they react with the biomolecules like nucleic acids, proteins and membrane lipids, hampering their normal functions. However, plants are equipped with antioxidant machinery that includes enzymatic and non-enzymatic antioxidants, such as superoxide dismutase (SOD), ascorbate peroxidase (APX), catalase (CAT), glutathione reductase (GR), ascorbic acid (AsA), α-tocopherols, glutathione and carotenoids. These antioxidants and antioxidant enzymes are up or down-regulated during stress and impart tolerance to plants [17]. Antioxidant activities increased in all organisms in large amounts and modulate physiological metabolisms in plants like transpiration, photosynthesis, respiration and nitrogen assimilation [17–18].

Calcium (Ca) is a macro-nutrient and has a crucial role in regulation of plant growth and development. It plays an imperative role in controlling the membrane structure and function [18–19]. A general idea is that Ca by binding to phospholipids, stabilizes lipid bilayers that consequently provide structural integrity to cellular membranes. Ca has been found to impede the negative effects of abiotic stress through the regulation of antioxidant metabolism and water relations [19–21]. However, the roles of Ca in regulation of heavy metal stress remains to be determined. Therefore, it would be of great importance to study whether an augment of Ca concentration would have an impact on mitigation of the toxic effects of heavy metals, such as Cd, on plant growth and development.

Indian mustard (*Brassica juncea* L.) belonging to Brassicaceae family is a very important oil seed crop. Mustard oil is one of the major edible oils in India and is having medicinal importance as well. Residual part of seeds is used as cattle feed and as fertilizer. Being a fast growing plant, it produces a high biomass even in heavy metal polluted soils. The present study was undertaken to investigate the effect of excessive Cd stress on the growth, oil content, biochemical aspects, Chl pigment contents and total protein content in *B. juncea*. More importantly, we provided insight into how Ca confers tolerance to mustard plants against Cd stress, demonstrating a new strategy to deal with heavy metal stress which can be used in agriculture as well as in removing metal from contaminated soils.

## Materials and Methods

### Plant materials and growth conditions

Seeds of *Brassica juncea* L. (Czern. & Coss.) were sown in earthen pots containing 5 kg of peat, perlite and sand (1:1:1, v/v/v) and Hoagland nutrient solution (200 mL pot^−1^) under glasshouse conditions. The Hoagland solution used in this experiment had the following composition (mg l^−1^): 270 N (KNO_3_), 31 P (KH_2_PO_4_), 234 K (KNO_3_), 200 Ca (Ca(NO_3_)_2_.4H_2_O), 64 S (MgSO_4_.7H_2_O), 48 Mg (MgSO_4_.7H_2_O), 2.8 Fe (Fe-EDTA), 0.5 Mn (MnCl_2_.4H_2_O), 0.5 B (H_3_BO_3_), 0.02 Cu (CuSO_4_), 0.05 Zn (ZnSO_4_.7H_2_O) and 0.01 Mo (H_3_MoO_4_.H_2_O) [22]. The pH of nutrient solution was adjusted to 6.5 with 0.1 mM KOH. After germination, 4-day-old seedlings were transferred to pots (one plant per pot) with the same ratio of peat, perlite and sand supplemented with nutrient solution (200 mL pot^−1^) and grown for an additional three weeks at average day/night temperatures of 28°C/15°C. Subsequently, 25-day-old plants were treated with different concentrations of Cd (CdSO_4_.8H_2_O) dissolved in nutrient solution with or without sprayed Ca (CaCl_2_) as below:

(1) Nutrient solution alone (control) (S0): 0.0 mg L^−l^ Cd + 0 mM Ca
(2) Ca alone (S1): 0 mg L^−l^ Cd + 50 mM Ca
(3) Cd stress alone (S2): 200 mg L^−l^ Cd + 0 mM Ca
(4) Cd stress and Ca (S3): 200 mg L^−l^ Cd + 50mM Ca
(5) Cd stress alone (S4): 300 mg L^−l^ Cd + 0 mM Ca
(6) Cd stress and Ca (S5): 300 mg L^−l^ Cd + 50mM Ca.

200 mL of Hoagland nutrient solution along with dissolved Cd was applied every alternate day to each pot except control which received only nutrient solution. To maintain the moisture content of the pot, 100 mL of distilled water was applied to each pot every day. CaCl_2_ (50 mM) was mixed with Tween-20, and sprayed to plants with a manual sprayer (10 mL plant^−1^) in the evening of every alternate day, from the 1^st^ day of treatment to day 90^th^. The experiment was laid out in a completely randomized design with five replicates. The plant samples were collected for analysis after 30 (55-day-old plants), 60 (85-day-old plants) and 90 (115-day-old plants) days after treatment (DAT). For determination of oil content, 25-, 55- and 85-day-old plants were subjected to S1, S2, S3, S4 and S5 treatments for 90 days (90 DAT), 60 days (60 DAT) and 30 days (30 DAT), respectively. Seeds were then collected from 115-day-old plants for oil extraction. Each treatment was replicated four times in a randomized block design and each replicate included 5 plants (i.e. 20 plants per treatment).

### Growth parameters

Growth parameters, including plant height, root length and plant dry weight (DW), were recorded at 30, 60 and 90 DAT.

### Estimation of oil content

Oil content was determined by following the solvent extraction technique in which 3 g of mustard seeds were crushed in Na_2_SO_4_ and the resultant powder containing oil was taken in test tubes. 20 mL of hexane was poured in the test tubes as mobile phase. Elute containing oil was stored in a vile and hexane was evaporated in hot water bath. The remaining oil was weighed and its percentage was calculated as follows: Oil percentage = oil content/seed weight × 100.

### Cadmium accumulation

The Cd contents in shoots and roots were determined using a Perkin-Elmer (Analyst Model 300) atomic absorption spectrophotometer. The heavy metal content was expressed as mg g^−l^ plant DW.

### Estimation of pigments

Chlorophyll content of the leaves was determined by the method proposed previously [23]. The absorbance was read at 663nm, 645nm and 480nm against 80% acetone used as a blank.

### Estimation of total protein content

Total protein content was estimated with Bradford method [24]. Absorbance was recorded spectrophotometrically at 595 nm (Beckman 640 D, USA) using bovine serum albumin as a standard.

### Estimation of proline content

Proline concentration was determined using a previously described method [25]. Absorbance was determined spectrophotometrically at 520 nm (Beckman 640 D, USA) using toluene as a blank.

### Extraction of the enzymes

Fresh leaves (10 g) were crushed in 50 volumes of 100 mM Tris-HCl (pH 7.5) containing 5 mM DTT (Dithiothreitol), 10 mM MgCl_2_, 1 mM EDTA (Ethylenediaminetetraacetic acid), 5 mM magnesium acetate, 1.5% PVP-40 (Polyvinylpyrrolidone), 1 mM PMSF (phenylmethanesulfonyl fluoride) and 1 μg mL^−1^ aproptinin. The homogenate was filtered using a cheese cloth and subjected to centrifugation for 15 min at 10,000 rpm. The source of enzymes was the supernatant collected after centrifugation. For the analysis of APX activity, tissues were separately homogenized with 2 mM AsA. All experiments were performed at 4°C.

### Enzyme assays

**Superoxide dismutase**. Activity of SOD (EC 1.15.1.1) was estimated according to [26] following the photoreduction of nitroblue tetrazolium (NBT). The activity of SOD was expressed as enzyme unit (EU) mg^−1^ protein. One unit of SOD was defined as the amount of protein causing 50% decrease of the SOD-inhibitable NBT reduction.

**Catalase**. Catalase (EC 1.11.1.6) activity was determined according to [27]. The activity of CAT was calculated using the extinction co-efficient of 36 × 103 mM^−l^ cm^−l^ and expressed as EU mg^−1^ protein.

**Ascorbate peroxidase**. The method of Nakano and Asada [28] was followed for the determination of APX activity. The decrease in absorbance was read at 265 nm. APX activity was expressed as EU mg^−l^ protein.

**Glutathione reductase**. The activity of GR (EC 1.6.4.2) was determined according to [29]. The decrease in absorbance was read at 340 nm for 2 min. The activity of GR was calculated using the extinction co-efficient of NADPH of 6.2 mM^−1^ cm^−1^ and expressed as EU mg^−l^ protein.

### Estimation of lipid peroxidation

Lipid peroxidation was determined by measuring the amount of malondialdehyde (MDA) produced by the thiobarbituric acid reaction as previously described [30]. Absorbance was recorded at 600 nm and the blank used was 1% thiobarbituric acid (TBA) in 20% trichloroacetic acid (TCA). The concentration of MDA was calculated using an extinction coefficient of 155 mM cm^−l^.

### Estimation of inorganic nutrients

Dried shoot and root materials (100 mg) were powdered and digested in H_2_SO_4_/HNO_3_ mixture (1/5, v/v) for 24 h, then were treated with HNO_3_/HClO_4_ mixture (5/1, v/v). The element concentration was measured by atomic absorption spectrophotometer (Analyst 300, Perkin-Elmer, Germany).

### Statistical analysis

One-way analysis of variance (ANOVA) was used for statistical analysis followed by Duncan’s Multiple Range Test (DMRT). The values obtained were the mean ± SE. for five replicates in each group. *P* values at 0.05 were considered as significant.

## Results

### Ca improved growth and biomass of mustard plants under Cd stress

Excessive Cd affected all the growth parameters of mustard plants that were examined, including plant height, root length and biomass per plant. The highest reduction in plant height was found to be 17.14%, 46.34% and 63.39%, respectively, after 30, 60 and 90 days of plant exposure to 300 mg L^−1^ Cd stress (S4) as compared with that of S0 control (Table 1). Treatment of mustard plants with 200 mg L^−1^ Cd (S2) showed lower decrease in plant height as compared with S4 treatment, and the decrease was 3.21%, 19.31% and 21.55% after 30, 60 and 90 DAT, respectively, over the untreated S0 control. Application of Ca to Cd-stressed plants reduced the negative effect of Cd on plant height. A decrease of only 16.17% and 56.14% was observed at S3 and S5, respectively, after 90 DAT when compared with that of the untreated control (S0). These data indicated that Ca could improve the height of mustard plants under Cd stress. Plants treated with Ca alone did not exhibit significant change in their height.

**Table 1:** Effect of Ca on growth of mustard plants under Cd stress.

Treatments	Plant height (cm)			Root length (cm)			Dry weight (g plant^−1^)		
	30 DAT	60 DAT	90 DAT	30DAT	60 DAT	90 DAT	30 DAT	60 DAT	90 DAT
S0	9.33±0.94a^$^	35.73±1.54a^£^	103.90±2.83a^¥^	4.13±0.41a^$^	9.46±1.03a^£^	16.06±1.31a^¥^	7.4±0.69a^$^	11.5±1.13a^£^	14.2±1.27a^¥^
S1	9.4 ±0.95a^$^	36.07±1.54a^£^	104.1±2.85a^¥^	4.9±0.44b^$^	10.1±1.09b^£^	16.86±1.36a^¥^	7.9±0.72a^$^	12.0±1.23a^£^	14.8±1.29a^¥^
S2	9.03±0.84b^$^	28.83±1.47b^£^	81.50±1.98b^¥^	3.50±0.34c^$^	7.20±0.67c^£^	12.1±1.16b^¥^	4.5±0.47b^$^	6.4±0.67b^£^	7.8±0.71b^¥^
S3	9.27±0.92c^$^	31.60±1.50c^£^	87.10±2.06c^¥^	3.79±0.35d^$^	8.49±0.88d^£^	13.95±1.22c^¥^	5.5±0.58c^$^	7.1±0.69c^£^	8.1±0.74c^¥^
S4	7.73±0.69d^$^	19.17±1.2d^£^	38.03±1.62d^¥^	2.45±0.23e^$^	5.37±0.46e^£^	8.8±0.98d^¥^	3.6±0.35d^$^	5.1±0.53d^£^	6.3±0.66d^¥^
S5	8.10±0.76e^$^	21.27±1.24e^£^	45.57±1.74e^¥^	3.1±0.27f^$^	6.77±0.57f^£^	10.25±1.12e^¥^	4.2±0.40b^$^	5.9±0.60b^£^	7.1±0.69e^¥^

Data presented are the means ± SE (n = 5). Different letters next to the number indicate significant difference (*P* < 0.05) among the treatments within a developmental stage. Symbols $, £ and ¥ denote significant change among the different developmental stages in the same treatment for the given parameter. S0 = Control, S1 = Cd (0 mg L^−1^) + Ca (50 mM), S2 = Cd (200 mg L^−1^), S3 = Cd (200 mg L^−1^) + Ca (50 mM), S4 = Cd (300 mg L^−1^), S5 = Cd (300 mg L^−1^) + Ca (50 mM). DAT, days after treatment.

Root length was decreased with increasing concentration of Cd. The S2 Cd treatment reduced the root length by 15.25%, 23.89% and 24.65% after 30, 60 and 90 DAT, respectively, relative to the S0 control (Table 1). The highest decrease in root length was noted at S4 concentration with 45.20% after 90 DAT. Treatment of Cd-stressed plants with Ca (S3) mitigated the negative effect of Cd stress on root length, showing only 8.23%, 10.25% and 13.13% decrease after 30, 60 and 90 DAT, respectively, over the S0 control. In case of S5, a positive effect of Ca application was also observed in root growth (36.17% over the untreated control at day 90) as compared with the S4 (45.20% over the untreated control at day 90). No significant increase in root length was recorded in plants treated with Ca alone when compared with that of S0.

Cd treatment of 200 mg l^−1^ (S2) decreased the plant DW by 39.18%, 44.34% and 45.07% after 30, 60 and 90 DAT, respectively, relative to the S0 control (Table 1). In comparison with the untreated control, the highest decrease in DW (55.63%) was observed after 90 day of plant exposure to Cd stress of 300 mg l^−1^ (S4). Application of Ca alone (S1) showed no significant change in plant DW. However, when Ca was added to Cd-stressed plants, improved plant DW was observed compared with Cd stress alone. S3 treatment showed an improved plant growth with only 25.67%, 38.26% and 42.95% decrease in plant DW over the S0 control after 30, 60 and 90 DAT, respectively. Similar improved tendency was observed when S4 and S5 treatments were compared. In the presence of Ca (S5), Cd-stressed plants exhibited better growth with only 43.24%, 48.69% and 50.0% decrease in DW after 30, 60 and 90 DAT, respectively, as compared with that of S4 treatment. There results collectively indicate that Ca plays an important role in mitigation of negative effect of Cd stress on plant growth and development.

### Ca moderated negative effect of Cd on oil production in mustard plants

**Fig 1:**
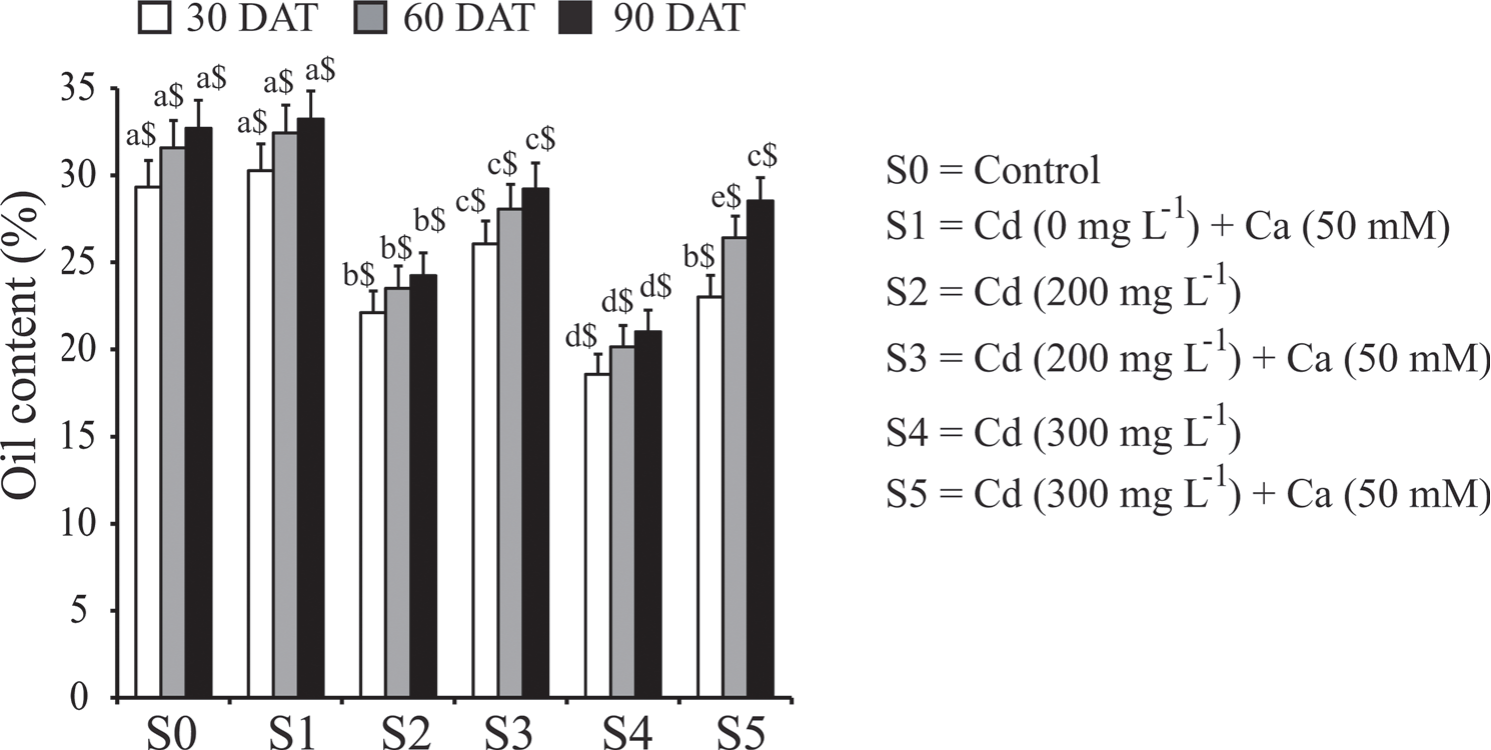
Effect of Ca on oil content in seeds of mustard plants under Cd stress at different time intervals. Data presented are the means ± SE (n = 5). Different letters indicate significant difference (*P* < 0.05) among the treatments within a developmental stage. Symbols $, £ and ¥ denote significant change among the different developmental stages in the same treatment. DAT, days after treatment.

Oil is an important product of mustard plants. Thus, it is important to examine the effect of Cd stress on oil content and the potential positive impact of Ca on Cd-stressed plants. Data shown in Fig. 1 indicated that Cd stress severely reduced the oil content. Specifically, the oil content was decreased to 24.11% and 21.55% at S2 and S4 concentrations, respectively, after 30 DAT from 29.33% of control S0 level. At 90 DAT of S2 and S4 concentrations, the oil content was decreased to 26.12% and 23.31%, respectively, from the control level of 32.71% (S0). However, supplementation of Ca with Cd showed enhanced oil content. For instance, at S5 treatment the oil content increased to 26.71% after 90 DAT, a 3.4% higher than that of respective S4 control (23.31%). This increase was significant, reflecting more than 10% increase of total oil content when compared with that of normal growing S0 control plants (32.71%). Non-significant change in oil content was observed on application of Ca to control plants. The data suggested that the oil percentage enhanced by Ca application on Cd stressed plants.

### Ca reduced Cd uptake to overcome Cd toxicity

To examine whether the positive impact of Ca treatment on Cd-stressed plants was associated with its ability to reduce plant’s Cd uptake, the Cd content in the plants subjected to Cd stress with and without Ca application was measured. As shown in Table 2, exogenous application of Ca reduced Cd accumulation in both shoots and roots as evidenced by 11.50% and 18.38% decrease in S3 and 31.77% and 36.72% decrease in S5 in comparison with the respective S2 and S4, respectively. This effect of Ca on minimizing the Cd uptake may at least partly contribute to the improved growth of mustard plants under Cd stress. In addition, we observed that the roots accumulated higher Cd than shoots at both the levels of Cd treatments (Table 2, S2 and S4).

**Table 2:** Effect of Ca on the Cd uptake in shoots and roots of mustard plants 90 days after treatment with Cd.

	S0	S1	S2	S3	S4	S5
Cd content in shoots (μM g^−1^ DW)	Nd	Nd	17.21±1.23a^$^	15.23±1.08b^$^	20.77±1.23c^$^	14.17±1.34d^$^
Cd content in roots (μM g^−1^ DW)	Nd	Nd	29.05±1.54a^£^	23.71±1.24b^£^	38.12±1.34c^£^	24.12±1.27b^£^

Data presented are the means ± SE (n = 5). Different letters next to the number indicate significant difference (*P* < 0.05) among the treatments within a developmental stage. Symbols $ and £ denote significant change between the shoots and roots in the same treatment. S0 = Control, S1 = Cd (0 mg L^−1^) + Ca (50 mM), S2 = Cd (200 mg L^−1^), S3 = Cd (200 mg L^−1^) + Ca (50 mM), S4 = Cd (300 mg L^−1^), S5 = Cd (300 mg L^−1^) + Ca (50 mM). Nd, not detected; DW, dry weight.

### Effect of Ca on pigment content of mustard plants under Cd stress

Cd adversely affected the pigment content in mustard plants as shown by the present study (Table 3). Chl *a* content was decreased by 47.36% at S2 concentration after 90 DAT as compared with S0 control. A decrease of 53.15% was observed with S4 treatment after 90 DAT as compared with untreated S0 control. Ca supplementation improved the Chl *a* content under both the Cd treatments. Only 26.31% and 49.47% reductions in Chl *a* content were noted at S3 and S5 concentrations, respectively, after 90 DAT when compared with the plants treated with Cd alone (S2 and S4). No significant change in Chl *a* level was observed with application of only Ca to plants (S1). As for the Chl *b*, its level was decreased by 25.25% and 34.34% at S2 and S4 concentrations, respectively, after 90 DAT over the S0 control (Table 3). Co-application of Ca and Cd showed lower decrease in Chl *b* content as compared with plants treated with Cd alone. An 18.18% and 27.27% reduction in Chl *b* content was observed only at S3 and S5 concentrations, respectively, after 90 DAT.

**Table 3:** Effect of Ca on chlorophyll (Chl) and carotenoid contents in leaves of mustard plants under Cd stress.

Treatments	Chl *a* (mg g^−l^ FW)			Chl *b* (mg g^−l^ FW)			Total Chl (mg g^−l^ FW)			Carotenoid (mg g^−l^ FW)		
	30 DAT	60 DAT	90 DAT	30 DAT	60 DAT	90 DAT	30 DAT	60 DAT	90 DAT	30 DAT	60 DAT	90 DAT
S0	1.3±0.91a^$^	1.7±0.92a^£^	1.9±0.8a^¥^	0.81±0.09a^$^	0.92±0.05a^£^	0.99±0.08a^¥^	2.11±0.33a^$^	2.62±0.32a^£^	2.89±0.90a^¥^	0.45±0.02a^$^	0.47±0.05a^£^	0.49±0.04a^¥^
S1	1.33±0.87a^$^	1.75±0.93a^£^	1.96±0.77a^¥^	0.82±0.05a^$^	0.93±0.07a^£^	0.99±0.06a^¥^	2.15±0.5a^$^	2.68±0.21a^£^	2.95±0.73a^¥^	0.48±0.03a^$^	0.52±0.07b^£^	0.57±0.02b^¥^
S2	0.88±0.08b^$^	0.95±0.81b^£^	1.0±0.9b^¥^	0.63±0.09b^$^	0.70±0.05b^£^	0.74±0.05b^¥^	1.51±0.62b^$^	1.65±0.53b^£^	1.74±0.75b^¥^	0.38±0.03b^$^	0.39±0.05c^£^	0.40±0.07c^¥^
S3	1.1±0.09a^$^	1.3±0.81c^£^	1.4±0.91c^¥^	0.69±0.08c^$^	0.77±0.06c^£^	0.81±0.05c^¥^	1.79±0.64c^$^	2.07±0.62c^£^	2.21±0.59c^¥^	0.42±0.04a^$^	0.43±0.04d^£^	0.44±0.02d^¥^
S4	0.72±0.07c^$^	0.81±0.05d^£^	0.89±0.08d^¥^	0.55±0.03d^$^	0.61±0.04d^£^	0.65±0.06d^¥^	1.27±0.45d^$^	1.42±0.43d^£^	1.54±0.61d^¥^	0.28±0.040c^$^	0.29±0.03e^£^	0.30±0.02e^¥^
S5	0.80±0.03d^$^	0.89±0.07e^£^	0.96±0.07e^¥^	0.61±0.03b^$^	0.68±0.04b^£^	0.72±0.08b^¥^	1.41±0.23e^$^	1.57±0.84e^£^	1.68±0.42e^¥^	0.33±0.02d^$^	0.34±0.02f^£^	0.35±0.02f^¥^

Data presented are the means ± SE (n = 5). Different letters next to the number indicate significant difference (*P* < 0.05) among the treatments within a developmental stage. Symbols $, £ and ¥ denote significant change among the different developmental stages in the same treatment. S0 = Control, S1 = Cd (0 mg L^−1^) + Ca (50 mM), S2 = Cd (200 mg L^−1^), S3 = Cd (200 mg L^−1^) + Ca (50 mM), S4 = Cd (300 mg L^−1^), S5 = Cd (300 mg L^−1^) + Ca (50 mM). DAT, days after treatment; FW, fresh weight.

Total Chl content was also decreased in mustard plants treated with increasing concentration of Cd (Table 3). S2 treatment showed less decrease in total Chl content with 39.79% as compared with S4 that triggered a 46.71% reduction after 90 DAT over the S0 control. Application of Ca and Cd together showed less effect on Chl production as compared with the plants treated alone with Cd. S3 and S5 treatments resulted in a decrease of only 23.52% and 41.86%, respectively, after 90 DAT. No significant increase was observed by the application of Ca to plants treated with Cd alone.

Carotenoid content was decreased by 15.55%, 17.02% and 18.36% in mustard plants treated with Cd for 30, 60 and 90 days, respectively, at S2 concentration (Table 3). Plants treated with S4 concentration showed a higher reduction in carotenoid content with 37.77%, 38.29% and 38.77%, respectively, after 30, 60 and 90 DAT as compared with the untreated S0 control. Plants supplemented with Ca alone (S1) exhibited insignificant increase in carotenoid content. Supplementation of Ca to Cd-stressed plants (S3 and S5) showed less decrease over the S0 control (only 10.20% and 30.61% decrease in S3 and S5 treatments, respectively, relative to the S0 control) in carotenoid level when compared with that observed in the plants treated alone with Cd (18.36% and 38.77% decrease in S2 and S4 treaments, respectively, relative to the S0 control) after 90 DAT.

### Effect of Ca on total protein content of mustard plants under Cd stress

The results related to the effect of Cd on total protein content of mustard plants in presence and absence of Ca was depicted in Table 4. Treatment of plants with Cd at S2 concentration decreased the protein content by 24.08%, 27.05% and 31.77% after 30, 60 and 90 DAT, respectively. A higher decrease in protein content of Cd-stressed plants was observed with 43.02%, 43.52% and 44.85% after 30, 60 and 90 DAT, respectively, with increasing Cd concentration (S4). Co-application of Ca and Cd at S5 concentration reduced the negative effect of Cd, showing lower decrease of 33.55%, 36.47% and 39.25% after 30, 60 and 90 DAT, respectively, over the S4 treatment.

**Table 4:** Effect of Ca on total protein content and proline content in leaves of mustard plants under Cd stress.

Treatments	Total protein content(mg g^−l^ FW)			Proline content(μg g^−l^ FW)		
	30DAT	60DAT	90DAT	30DAT	60DAT	90DAT
S0	6.02±0.60a^$^	8.5±0.79a^£^	10.7±1.02a^¥^	51±2.51a^$^	54±2.55a^£^	55±2.56a^¥^
S1	6.5±0.68b^$^	9.1±0.86b^£^	11.2±1.12b^¥^	51.2±2.51a^$^	54.4±2.56a^£^	55.2±2.57a^¥^
S2	4.57±0.48c^$^	6.2±0.65c^£^	7.3±0.70c^¥^	93±2.87b^$^	125±3.31b^£^	159±4.05b^¥^
S3	5.1±0.53d^$^	6.7±0.68d^£^	8.0±0.74d^¥^	107±3.1c^$^	132±3.36c^£^	167±4.20c^¥^
S4	3.43±0.33e^$^	4.8±0.50e^£^	5.9±0.61e^¥^	115±3.3d^$^	143±3.49d^£^	187±4.33d^¥^
S5	4.0±0.42f^$^	5.4±0.57f^£^	6.5±0.67f^¥^	130±3.6e^$^	153±3.91e^£^	194±4.58e^¥^

Data presented are the means ± SE (n = 5). Different letters next to the number indicate significant difference (*P* < 0.05) among the treatments within a developmental stage. Symbols $, £ and ¥ denote significant change among the different developmental stages in the same treatment. S0 = Control, S1 = Cd (0 mg L^−1^) + Ca (50 mM), S2 = Cd (200 mg L^−1^), S3 = Cd (200 mg L^−1^) + Ca (50 mM), S4 = Cd (300 mg L^−1^), S5 = Cd (300 mg L^−1^) + Ca (50 mM). DAT, days after treatment; FW, fresh weight.

### Ca reduced lipid peroxidation in Cd-stressed mustard plants

**Fig 2:**
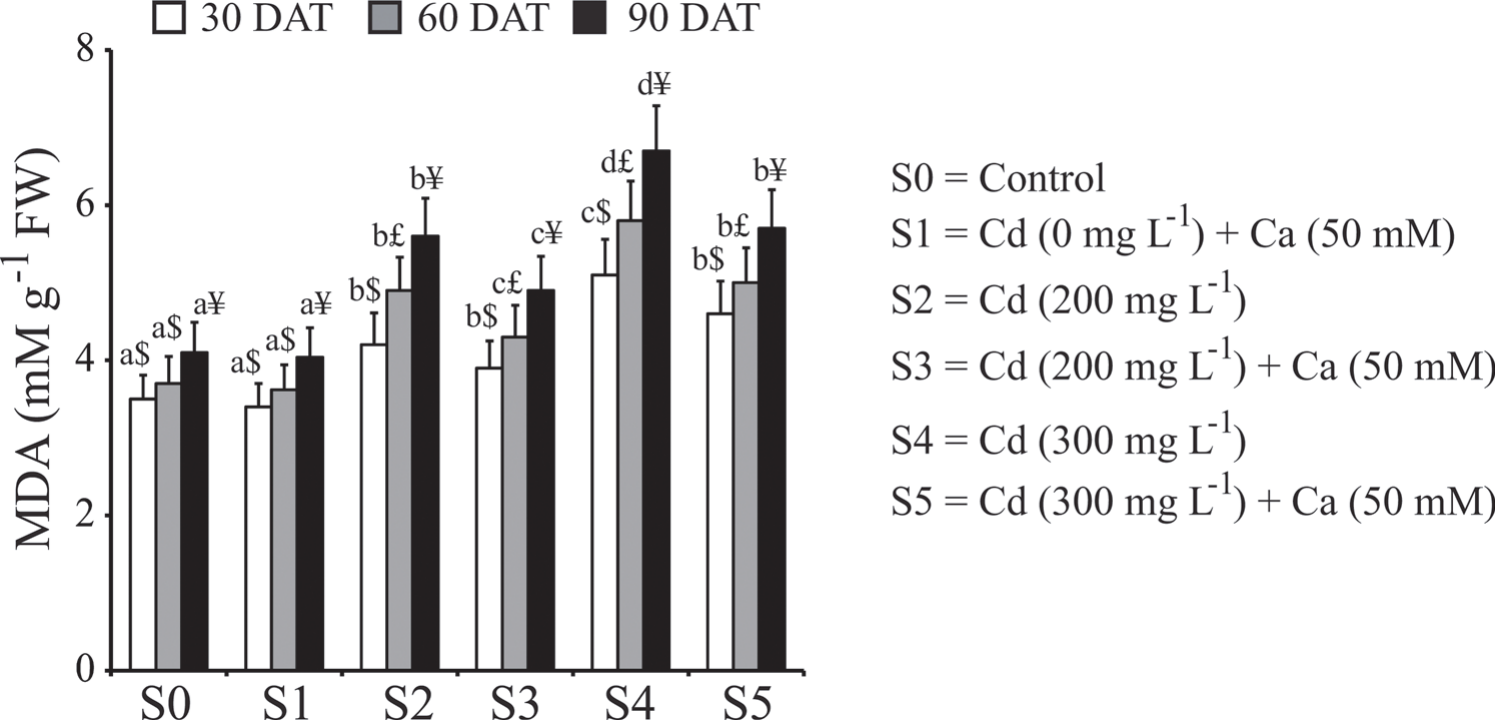
Effect of Ca on malondialdehyde (MDA) content in leaves of mustard plants under Cd stress at different stages of growth. Data presented are the means ± SE (n = 5). Different letters indicate significant difference (*P* < 0.05) among the treatments within a developmental stage. Symbols $, £ and ¥ denote significant change among the different developmental stage in the same treatment. DAT, days after treatment; FW, fresh weight.

The content of MDA, a product of lipid peroxidation, was determined in mustard plants subjected to various treatments to assess the degree of membrane damage resulted from Cd stress and the effect of Ca in reducing such negative damage. As shown in Fig. 2 The Cd stress increased the MDA content in mustard plants by 16.66%, 24.48% and 26.78% after 30, 60 and 90 DAT, respectively, at S2 concentration as compared with the S0 control. A further increase in MDA content was observed with increasing Cd concentration in stress treatment (S4) with 31.37%, 36.20% and 38.80% after 30, 60 and 90 DAT relative to the untreated S0 control. When Ca was supplemented to Cd-stressed plants, significantly lower accumulation of MDA was recorded. Specifically, only a 13.95% and 16.32% accumulation was observed in S3 treatment, and 26.00% and 28.07% accumulation was noted for S5 treatment after 60 and 90 DAT, respectively. These data might suggest a healing effect of Ca for membrane of mustard plants under Cd stress. Control plants supplemented with Ca alone exhibited no significant change in MDA content.

### Ca enhanced proline biosynthesis in Cd-stressed mustard plants

Proline has been known as an osmoprotectant and antioxidant, playing important role in stress management in plants [31]. Its titer is correlated with the ability of plants to tolerate environmental stress to which they are exposed [32,33]. Mustard plants treated with Cd at concentration of 200 mg l^−1^ (S2) showed an increase in proline content by 45.16%, 56.80%, 65.40% after 30, 60 and 90 DAT, respectively, over the S0 untreated control (Table 4). With increasing Cd concentration used in treatment (S4), further increase in proline content was observed in treated plants; specifically, 55.65%; 62.23% and 70.58% increase was found after 30, 60 and 90 DAT, respectively. These data indicated that proline biosynthesis was significantly enhanced in mustard plants in response to Cd stress. Supplementation of Ca along with Cd further enhanced the proline content. For instance, S3 treatment showed an increase in proline content by 52.33%, 59.09% and 67.06%, whereas S5 treatment exhibited 60.76%, 64.70% and 71.64% enhancement relative to the S0 control after 30, 60 and 90 DAT, respectively, which are significantly higher than their respective Cd treatment alone (Table 4, S2 and S4). This result suggests a positive effect of Ca on mustard response to Cd stress in terms of proline biosynthesis. No significant change was observed in proline content by application of Ca alone to the mustard plants.

### Ca modulates antioxidant enzymes to protect mustard plants under Cd stress

**Fig 3:**
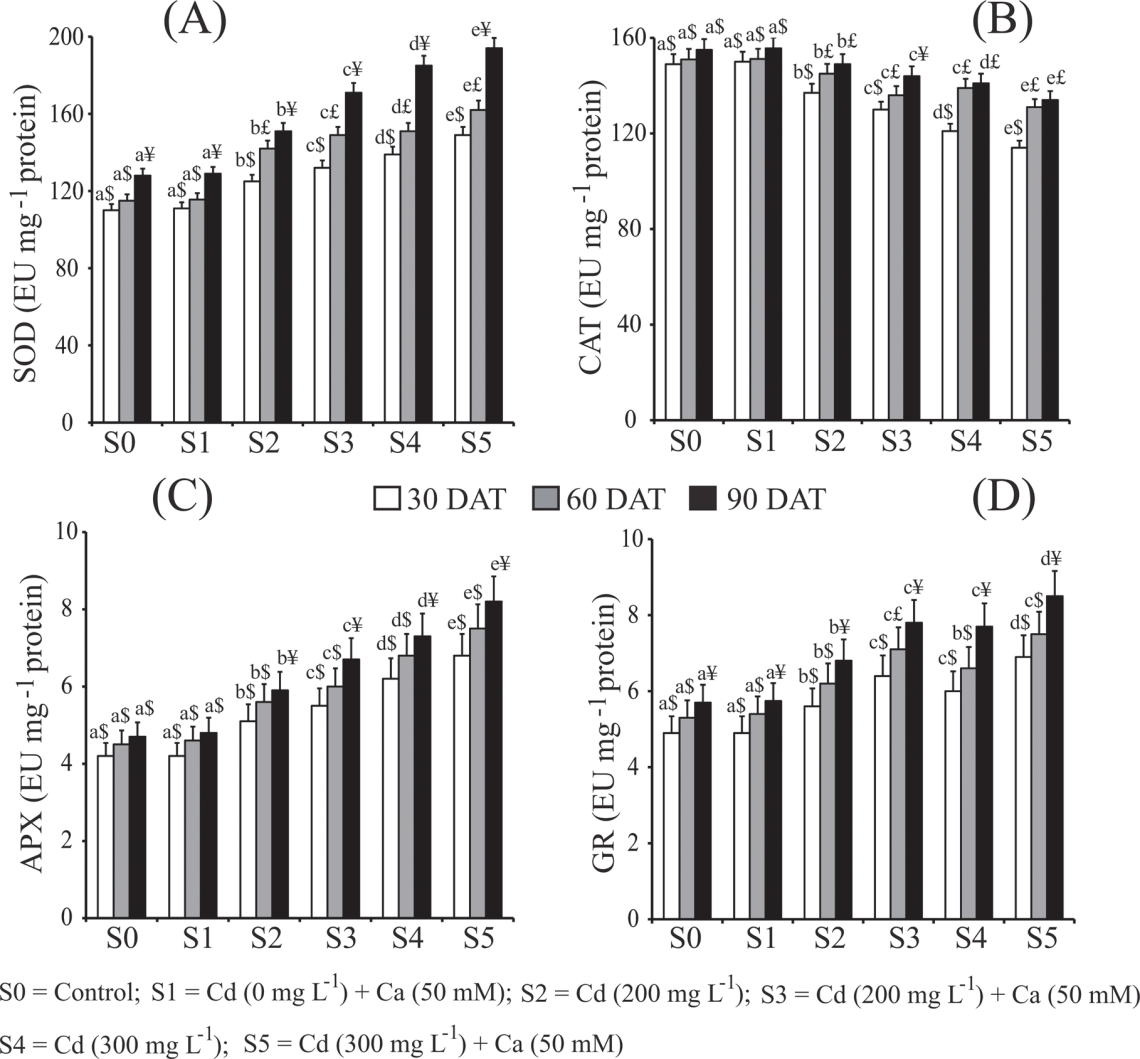
Effect of Ca on (A) superoxide dismutase (SOD); (B) catalase (CAT), (C) ascorbate peroxidase (APX); and (D) glutathione reductase (GR) in leaves of mustard plants under Cd stress at different stages of growth. Data presented are the means ± SE (n = 5). Different letters indicate significant difference (*P* < 0.05) among the treatments within a developmental stage. Symbols $, £, ¥ denote significant change among the different developmental stage in the same treatment. DAT, days after treatment.

Various assays were also performed to evaluate the activity of a number of antioxidant enzymes under Cd stress. SOD activity was increased with increasing concentration of Cd (Fig. 3A). After 30 DAT the increase was 12.00% and 20.86% at S2 and S4 concentrations, respectively. A higher increase with 15.23% and 30.81% at S2 and S4 concentrations was also observed after 90 DAT relative to the S0 control. Application of Ca in presence of Cd showed a further increase of 25.14% and 34.02% in SOD activity at S3 and S5 concentrations, respectively, after 90 DAT.

The results pertaining to the effect of Cd in presence and absence of Ca on CAT activity in mustard plants was given in Fig. 3B. CAT activity was decreased as the concentration of Cd increased. The highest decrease of 18.79% in CAT activity was observed at S4 concentration after 30 DAT in comparison with the S0 control. The lowest increase was recorded after 60 and 90 DAT. Ca added alone to control plants (S1) showed no significant change in CAT activity at all developmental stages. However, further decrease of 12.75% and 23.48% in CAT activity was observed when Ca was supplemented to Cd-stressed plants at S3 and S5 concentrations, respectively after 30 DAT.

As for the APX (Fig. 3C), after 90 DAT the minimum increase in APX activity was found to be 20.33% at S2 concentration, whereas the maximum increase was 35.61% at S4 concentration as compared with that of the S0 control. Application of Ca to Cd-stressed plants further increased the APX activity by 38.23%, 40.00% and 42.68% after 30, 60 and 90 DAT, respectively, at S5 concentration, while no significant increase was observed when Ca was applied alone to the control plant (S1).

Regarding GR activity, an increase by 12.50%, 14.51% and 16.17% after 30, 60 and 90 DAT, respectively, was observed at S2 concentration over the S0 control. Further increase in GR activity was noted at S4 concentration, with 19.67%, 22.05% and 25.97% after 30, 60 and 90 DAT, respectively, as compared to that of untreated control. The highest increase of 32.94% in GR activity was recorded over the control when Ca was supplied along with Cd to mustard plants (S5) after 90 DAT. Ca applied to plants alone did not show any significant change in GR activity compared with that of the untreated control at any time point (Fig. 3D).

### Ca enhanced the uptake of essential mineral elements in Cd-stressed mustard plants

Cd stress decreased the accumulation of essential nutrients in shoots as well as in roots of the mustard plants (Table 5). In shoots, S, Mn, Mg, Ca and K contents were decreased by 12.59%, 47.73%, 26.71%, 21.25% and 22.09%, respectively, at S2 concentration of Cd relative to the S0 control after 90 DAT. Increasing Cd concentration (S4) showed further decrease of 26.88%, 63.76%, 36.48%, 31.49% and 48.95% in S, Mn, Mg, Ca and K content of shoots, respectively, over the control. Application of Ca was able to alleviate the negative effect of Cd stress on ion accumulation. A lower decrease of only 21.48%, 59.58%, 29.31%, 26.77% and 31.74% was observed in S, Mn, Mg, Ca and K content of shoots, respectively, at S5 concentration relative to S0 control, clearly indicating the improving effect of Ca on the accumulation of essential nutrients in Cd-stressed plants. Similar phenomenon was observed in accumulation of these examined elements in roots. The contents of all the examined elements decreased by Cd treatments alone (S2 and S4) in roots which partially recovered when Ca was co-applied with Cd to the plants (S3 and S5) (Table 5).

**Table 5:** Effect of Ca on the mineral element contents in shoots and roots of mustard plants 90 days after treatment with Cd.

Mineral nutrition(μM g^−1^ DW)	S0	S1	S2	S3	S4	S5
Shoot S	135±3.7a^$^	135.5±3.7a^$^	118±3.3b^$^	125±3.35c^$^	98.7±2.92d^$^	106±3.1e^$^
Shoot Mn	28.7±1.43a^$^	29.6±1.45a^$^	15±1.30b^$^	19.3±1.33c^$^	10.4±1.08d^$^	11.6±1.14e^$^
Shoot Mg	307±5.34a^$^	310±5.36a^$^	225±4.5b^$^	255±4.96c^$^	195±4.0d^$^	217±4.34e^$^
Shoot Ca	127±3.42a^$^	128±3.43a^$^	100±2.96b^$^	115±3.2c^$^	87±2.72d^$^	93±2.84e^$^
Shoot K	715±9.4a^$^	717±9.48a^$^	557±7.4b^$^	631±8.7c^$^	365±6.59d^$^	488±6.98e^$^
Root S	401±6.0a^£^	405±6.04a^£^	290±5.2b^£^	395±6.87c^£^	206±4.2d^£^	310±5.35e^£^
Root Mn	94±2.89a^£^	95±2.90a^£^	97±2.91a^£^	89±2.74b^£^	101±2.95c^£^	92±2.81a^£^
Root Mg	987±11.4a^£^	991±11.5a^£^	910±10.51b^£^	954±10.72c^£^	853±9.4d^£^	903±9.91b^£^
Root Ca	203±4.1a^£^	207±4.3a^£^	199±4.0b^£^	212±4.29c^£^	192±3.9d^£^	205±4.2a^£^
Root K	1510±13.3a^£^	1530±13.5a^£^	1275±11.7b^£^	1320±12.9c^£^	1030±11.8d^£^	1350±13.1c^£^

Data presented are the means ± SE (n = 5). Different letters next to the number indicate significant difference (*P* < 0.05) among the treatments within a developmental stage. Symbols $ and £ denote significant change between the shoots and roots in the same treatment. S0 = Control, S1 = Cd (0 mg L^−1^) + Ca (50 mM), S2 = Cd (200 mg L^−1^), S3 = Cd (200 mg L^−1^) + Ca (50 mM), S4 = Cd (300 mg L^−1^), S5 = Cd (300 mg L^−1^) + Ca (50 mM).; DW, dry weight.

## Discussion

Ca regulates a range of activities within the cell, such as cell division and elongation, cytoplasmic streaming, photomorphogenesis and plant defense against environmental stresses [34–37]. It has been established that Ca functions as the central node in overall signaling web and has a promising role in stress tolerance [38]. Ca stimulates calmodulin-like proteins that interact with Ca^2+^ ions. Changing their conformation in response to Ca-binding, calmodulin proteins regulate a variety of mechanisms, including ion transport, gene regulation, cell motility, growth, proliferation, apoptosis and stress tolerance [39–41].

In this study, we found that Ca application mitigated the negative effect of Cd stress on mustard plants, including their growth and oil content. Under our experimental conditions, the higher the Cd concentration was applied the higher the suppression of plant growth, in terms of plant height, root length and DW, was observed (Table 1), which is in agreement with the results reported in other plant species [11,42]. In plants, roots are the foremost organs which come in contact with the toxic metals and usually accumulate a higher amount of the metal than shoots [11,16]. Indeed, we observed higher Cd accumulation in roots than in shoots of mustard plants under Cd stress (Table 2). However, when Ca was co-applied with Cd, a significant decrease in Cd accumulation was observed in Cd-stressed mustard plants (Table 2), resulting in a significant increase of the growth parameters of the stressed plants (Table 1). The biosynthesis of oil, the major product of mustard plants, was severely affected by Cd stress as well (Fig. 1). Treatment of mustard plants with Cd caused remarkable decrease in oil content, whereas spraying Ca could significantly enhance the oil content in Cd-stressed plants. These results suggested that Cd inhibits the fatty acid biosynthesis machinery but not the fatty acid profile in mustard plants.

The mechanism of Cd toxicity is not completely understood yet. According to the published literature, Cd can reduce soil microbes, damage root tips, reduce nutrient and water uptake by plants and impair photosynthesis, leading to growth inhibition of plants [43–45]. Additionally, the growth inhibition by the Cd treatments may also result from inhibition in cell division and elongation rate of cells that mainly occur by an irreversible obstruction of proton pump responsible for the process [6,46]. The basis of these dysfunctions is due to the formation of metal thiolate bond and alteration of cell wall and membrane permeability by binding of nucleophylic groups [47–48]. The results of the current study suggested that the inhibition of mustard plant growth by Cd stress was associated with reduced photosynthesis. Indeed, data shown in Table 3 indicated that both Chl and carotenoid syntheses were negatively affected by Cd stress. The decline in Chl contents in mustard plants exposed to Cd stress might be due to (i) the inhibition of the synthesis of important enzymes, such as δ-aminolevulinic acid dehydratase and protochlorophyllide reductase, which are involved in Chl biosynthesis and (ii) impairment in the supply of Mg^2+^, Fe^2+,^ Zn^2+^ and Mg^2+^ that are required for the synthesis of Chl [49–51]. In fact, the decrease in mineral element uptake shown in Table 5 might contribute to reduced total Chl and an overall lower performance of mustard plants under Cd stress. On the other hand, carotenoids play a pivotal role in photo protection of chlorophylls against photo oxidative damage by quenching ROS, such as singlet oxygen [52]. Thus, being an efficient free-radical scavenger, a decrease in carotenoid content in plants subjected to heavy metal stresses, including Cd stress, may result in an overproduction of ROS that subsequently inhibits plant growth by causing oxidative damages to DNA, RNA and proteins [32,53]. Consistent with this observation, the protein content was declined in the Cd-stressed mustard plants (Table 4).

In addition, our data showed that the content of MDA, a product of lipid peroxidation, was increased by Cd treatment in mustard plants (Fig. 2), which has been widely known to cause cell membrane damage, as well as protein modification that may lead to reduced protein content [54–55]. Alternatively, the decrease in protein content in Cd-stressed plants might result from enhanced protein degradation process as a result of increased protease activity [56]. Our results clearly demonstrated that application of Ca decreased the MDA level (Fig. 2), enhanced Chl, carotenoid and protein biosynthesis (Tables 3 and 4) in Cd-stressed plants, leading to the overall improvement of growth of mustard plants under Cd stress (Table 1). This improved growth of Cd-stressed mustard plants may be connected with potential role of Ca in stabilizing membranes as Ca has been known to be responsible for decreased thiobarbutric acid and H_2_O_2_ content [19,57–58]. The pronounced effects of Ca on the improvement of growth under Cd stress may also be due to its role in enhancement of the essential mineral element uptake, which was impaired by Cd stress. Results of the present study indicated a significant decrease in the uptake of the examined elements by Cd stress in both shoots and roots of mustard plants. However, exogenous application of Ca remarkably alleviated the negative effect of Cd on the uptake of these nutrients (Table 5).

Proline is known to be accumulated and acts as an osmoprotectant and antioxidant to protect the plants under various environmental stresses [11,31,59]. Our data showed an increased proline accumulation by Cd stress as well (Table 4). Foliar application of Ca in combination with Cd was found to be effective in further increasing the accumulation of proline in Cd-stressed mustard plants, suggesting that Ca could enhance proline biosynthesis which is beneficial for the plants to withstand the Cd toxicity. Additionally, the activities of antioxidant enzymes examined, specifically SOD, APX and GR, were also enhanced in mustard plants by Cd stress, and these activities were further increased by spraying Ca to Cd-stressed mustard plants (Fig. 3). This result suggests that the enhanced activities of these enzymes are beneficial for responses of mustard plants to Cd stress and that Ca is able to stimulate the antioxidant systems related to these enzymes, which further strengths the mustard plants to withstand excessive Cd stress. During oxidative stress, SOD is considered as the first line of defense against ROS, which catalyzes the disproportionation of O_2_·ˉ to H_2_O_2_ and O_2_. It removes O_2_·ˉ, thus decreasing the risk of OH· formation from O_2_·ˉ via the metal-catalyzed Haber-Weiss-type reaction [60–61]. APX is a very important scavenger of H_2_O_2_ in water-water and ascorbate-glutathione cycles and utilizes AsA as the electron donor [62], while increase in the GR activity in plants results in the accumulation of glutathione levels and ultimately confers tolerance to plants [63]. On the other hand, the CAT activity was found to be reduced in Cd-stressed plants, and it was further reduced when Ca was applied (Fig. 3B). The function of CAT in elimination of H_2_O_2_ might be overcome by the increase in APX activity (Fig. 3C). It is well established that CAT and APX have common function in eliminating H_2_O_2_, but CAT has a much lower affinity for H_2_O_2_ than APX, and thus APX comes in to play when maximum amount of H_2_O_2_ is to be removed [17,55]. Supplementation of Ca increased the activity of APX but not that of CAT perhaps because maximum amount of H_2_O_2_ was eliminated by APX due to its higher affinity for H_2_O_2_ as compared with CAT.

In conclusion, Cd is a toxic metal and its negative effect on plant growth and development is increasing day by day. Application of Ca enhances Cd tolerance in mustard plants and significantly restores the morphological and biochemical attributes. Ca also alleviates the negative effect of oxidative stress induced by Cd. The activities of several enzymatic antioxidants (SOD, APX and GR) increased under Cd stress, and further increase was observed with co-application of Ca, suggesting that Ca enables the plants to withstand the Cd toxicity by increasing the activities of the antioxidant enzymes. Ca also significantly improved the uptake of mineral nutritional elements in Cd-stressed mustard plants, which might contribute to their enhanced photosynthesis. Therefore, fertilization with Ca can alleviate the negative effects of Cd stress and can help the plant enhance the metabolism to flourish better under the stressful conditions as evidenced by the enhancement of oil content in Cd-stressed mustard plants by treatment with Ca. It would be now of great interest to study the mechanism(s) by which Ca regulates plant responses to Cd stress by omic strategies. For instance, transcriptome analysis at whole genome level using RNA-seq could be carried out to discover the Ca-mediated pathways involved in regulation of plant adaptation to Cd stress. Potential candidate genes identified through transcriptome analysis could be then subjected to in-depth functional analyses prior to using them in genetic engineering for development of improved Cd stress-tolerant plants.
